# Bridging gaps in ophthalmology residency programs: the link between practice, training and confidence in ocular examination and gonioscopy for diagnosing glaucoma, a blinding disease

**DOI:** 10.1186/s12909-024-05665-y

**Published:** 2024-06-21

**Authors:** Ortal Fogel Tempelhof, Daphna Mezad-Koursh, Assaf Hilely, Dan Gaton, Shimon Kurtz

**Affiliations:** 1 Department of Ophthalmology, Sourasky Medical Center, Tel Aviv, Israel, affiliated to The Faculty of Medicine, Tel Aviv University, Tel Aviv, Israel; 2 Department of Ophthalmology, Rabin Medical Center, Petah-Tikva, Israel, affiliated to The Faculty of Medicine, Tel Aviv University, Tel Aviv, Israel

**Keywords:** Ophthalmology teaching, Glaucoma, Gonioscopy, Residency curriculum

## Abstract

**Background:**

To evaluate real-world utilization of gonioscopy for diagnosing glaucoma among ophthalmologists with diverse subspecialties, and understand current perceptions of teaching, training, and confidence in gonioscopy.

**Methods:**

A nationwide anonymous online survey was conducted among practicing ophthalmologists, querying about demographics, professional experience, practice of routine ocular examination for glaucoma and perceptions of confidence in performing them.

**Results:**

136 ophthalmologists participated in the survey, with various levels of experience from residency to over twenty years of ophthalmology practice. Glaucoma specialists comprised 23 (16.9%) of the participants. Of the non-glaucoma-specialist respondents, only 33 (29.2%) expressed being highly confident in interpreting gonioscopic findings, which correlated significantly with their self-reported inadequate level of training in gonioscopy during residency (*p* < 0.001) and even more so with their low frequency of implementing gonioscopy in routine examinations (*p* < 0.001). The commonly cited reasons for the low practice of gonioscopy were insufficient time allotted to examinations and lack of experience, knowledge or equipment.

**Conclusions:**

Gonioscopy is fundamental to the detection of glaucoma. This study reveals underutilization of gonioscopy in the practice of ophthalmology and its association with lower training opportunities, calling for expedited changes in the residency’s curriculum, alongside measures to promote its use in clinical practice.

## Introduction

**Fig. 1:**
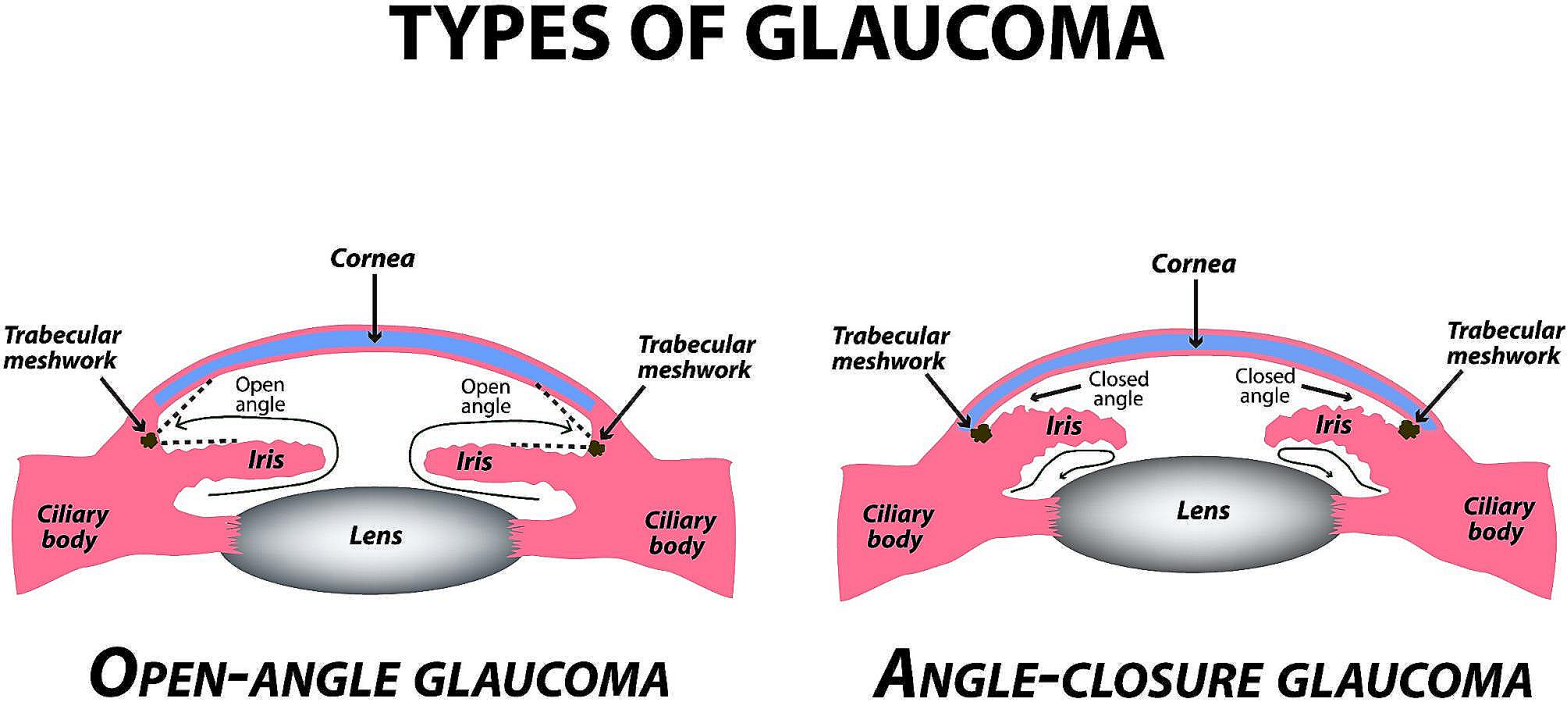
Schematic representation of open-angle glaucoma (left) and angle-closure glaucoma (right), highlighting key anatomical and pathological features. Copyrights: mikrostoker © 123RF.com

Glaucoma is a progressive optic neuropathy and a leading cause of irreversible blindness [1]. It was reported to affect more than 65 million people worldwide in 2013, and is expected to rise to 111.8 million by 2040 [2]. The two most common types of glaucoma, primary open-angle glaucoma (POAG) and primary angle-closure glaucoma (PACG), differ in the extent of accessibility to the anterior chamber angle (ACA) structures, the main drainage of the aqueous in the eye. (Fig. 1) Differentiating between the various glaucoma types is crucial since each mandates a different treatment approach, and the need for early accurate diagnosis is indisputable. POAG is estimated to be present in 3.05% of the world’s population compared to a presence of 0.5% for PACG, although the prevalence of various forms of glaucoma varies across different geographic regions, ethnicities, and sex [1–3]. Refraction errors were found to be associated with glaucoma, with hyperopia having been shown to be a risk factor for angle-closure glaucoma [4]. 

Gonioscopy is a slit-lamp examination, which includes the placement of a special lens containing mirrors or prisms on the cornea, allowing for the visualization and assessment of the ACA and any associated pathologies within the routine office ophthalmology examination. Gonioscopy remains one of the most valuable tools for differentiating among the various types of glaucoma and associated diseases and for diagnosing the patients at risk for developing glaucoma or an acute attack of substantial intraocular pressure (IOP) increase (i.e., acute angle closure glaucoma [AACG]). Despite its relative ease and speed in performance as well as its widespread accessibility, the technique requires training and practice to be skillfully executed. The ocular examination for glaucoma includes the assessment of the anterior segment (AS) of the eye, IOP, gonioscopy, and optic nerve head (ONH). It also requires understanding of the underlying mechanism of glaucoma and its association with any findings in the examination. For example, the ophthalmologist must be cognizant of the well-documented association between hyperopia, short AC depth, narrower ACA, and PACG [5]. The sequence of the examination is also crucial for accurate interpretation of the results, since, for instance, pupil dilation and pressure on the cornea from the goniolens might spuriously change the measured IOP and ACA configuration [6–12]. 

Our study aims to evaluate real-world utilization of gonioscopy in the daily practice, examine ophthalmologists’ perceptions of their training in gonioscopy and identify the obstacles impeding its widespread utilization, coupled with suggested strategies for promoting its wider use in residency and after.

## Methods

This study was approved by the Institutional Review Board (IRB TLV-0233-22) of the Tel Aviv Sourasky Medical Center and performed in accordance with the declaration of Helsinki. The survey incorporated a statement that willingly completing the anonymized survey would be equivalent to granting informed consent. The ethics committee therefore waived the requirement for a signed informed consent. The online survey tool was sent between September and December 2022 to all practicing ophthalmologists in Israel, at any level of training or experience, who were registered with the National Ophthalmology Society.

The survey was not sponsored by any pharmaceutical company and no financial incentive was given in exchange for participation. The survey was anonymous, and the responses were downloaded to a Microsoft Excel spreadsheet (Microsoft Corporation, 2016) and given consecutive numbers to maintain anonymity.

The questionnaire included 18 items of varying formats: open-ended questions and comments, and close-ended questions with either single- or multiple-choice answers for demographics, ophthalmology background (country of residency completion, subspecialty, years of experience since residency completion), and work venues (geographic region of employment and whether practicing in a hospital, a community clinic, or in a private setting). Further questions to be answered on a Likert scale dealt with the respondents’ perceptions of their professional training and level of confidence in various ocular examinations. The respondents were further queried about the technique by which they assessed the AS, IOP and gonioscopy. Finally, the survey featured a multiple-choice question querying reasons for the estimated low utilization of gonioscopy among ophthalmologists.

### Statistical analysis

Internal consistency was evaluated by Cronbach’s alpha. Continuous variables were presented as mean ± standard deviation and range. The associations between categorical variables were evaluated with the Chi-square test and those between ordinal variables with Spearman’s rank correlation coefficient. The association between categorical and ordinal variables was assessed using the Mann-Whitney test. All statistical tests were two-sided, and a *p* value of < 0.05 was considered statistically significant. SPSS software was used for all statistical analyses (IBM SPSS Statistics for Windows, version 28, IBM Corp, Armonk, New York, USA, 2021).

## Results

### Survey participants

**Fig. 2:**
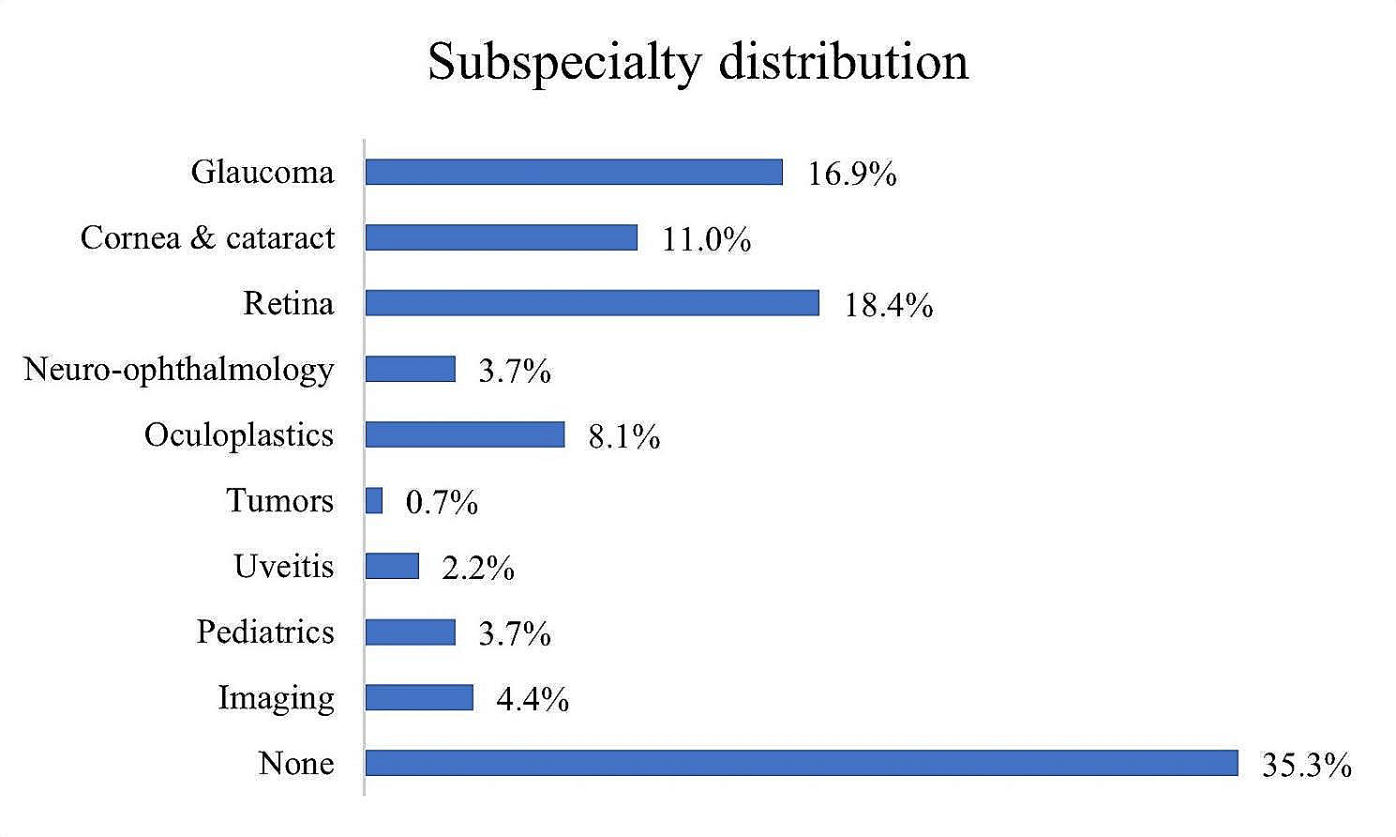
Subspecialty distribution in percentage. 4.4% of the responding ophthalmologists reported having more than one subspecialty

A total of 136 ophthalmologists, representing approximately 25% of the practicing ophthalmologists in the country, completed the survey. They were comprised of 70 (51.5%) males and 66 (48.5%) females, with a mean age of 50.7 ± 12.5 years (range 33–80 years). The vast majority of participants (*n* = 114, 83.8%) had completed their ophthalmology residency program in Israel, and current residents constituted 15 (11%) of the respondent pool. Approximately one-third of the respondents (*n* = 43, 31.6%) had completed their residency within the last decade, and another third (*n* = 50, 36.8%) had over 20 years of post-residency experience. Eighty-eight respondents (64.7%) had pursued a subspecialty, with 6 (4.4%) pursuing more than one (Fig. 2). 23 (16.9%) of the respondents identified themselves as glaucoma specialists (GS). Almost one-half of the respondents (*n* = 67, 49.3%) reported practicing in more than one workplace: 70% in hospital clinics, 60% in community clinics, and 33% in private clinics.

### Gonioscopy training during residency

The respondents’ estimates of the adequacy of their gonioscopy training during residency are summarized in Table 1. Fifty-seven (41.9%) reported having had from occasional to no training at all. A sub-analysis of GS and respondents who were not GS ([NGS], *n* = 113) revealed that the GS felt they received insufficient training compared to the NGS participants (*p* = 0.014). We attribute this difference to the glaucoma fellowship completed by GS, which highlights the inadequate gonioscopy training provided during residency.

**Table 1:** Respondents’ estimates of gonioscopy training during residency, distributed by glaucoma-specialist and non-glaucoma-specialist respondents

Gonioscopy training frequency (Likert scale)	Glaucoma specialist respondents, *n* = 23	Non-glaucoma- specialist respondents, *n* = 113	*p* value	Total, *n* = 136
Never (1) Rare (2) Occasional (3) Frequent (4) Very frequent (5)	2 (8.7%) 9 (39.1%) 3 (13.0%) 9 (39.1%) 0	5 (4.4%) 20 (17.7%) 18 (15.9%) 63 (55.8%) 7 (6.2%)	0.014	7 (5.1%) 29 (21.3%) 21 (15.4%) 72 (52.9%) 7 (5.1%)
Scale (mean ± SD)	2.83 ± 1.07	3.42 ± 0.99		3.32 ± 1.03

* SD = standard deviation

### Level of confidence in performing various ocular examinations and correlation to training in residency

The results of the participants’ grading of their level of confidence in performing various ocular examinations are summarized in Table 2. A vast majority of the NGS respondents reported being from highly to extremely confident in assessing AC depth (*n* = 98, 86.7%), IOP (*n* = 110, 97.3%), and ONH (*n* = 108, 95.6%). However, only 33 of them (29.2%) reported feeling highly to extremely confident in evaluating the ACA (structure and pigment) with a goniolens and 14 (12.4%) in performing dynamic gonioscopy. In this subgroup, confidence in gonioscopy and dynamic gonioscopy correlated significantly to the level of perceived gonioscopy training in residency (*p* < 0.001 and *p* = 0.007, respectively), but not to years of experience post-residency (*p* = 0.760 and *p* = 0.195, respectively).

**Table 2:** Respondents’ mean confidence levela in performing various ocular examinations, distributed by glaucoma-specialist and non-glaucoma-specialist respondents

Ocular examination (mean ± SD)	Glaucoma specialist respondents, n = 23	Non-glaucoma- specialist respondents, n = 113	*P* value	Total, n = 136
Anterior chamber depth	4.57 ± 0.51	4.23 ± 0.71	0.041	4.29 ± 0.69
Gonioscopy (structure & pigment)	4.57 ± 0.50	2.93 ± 0.96	< 0.001	3.21 ± 1.09
Dynamic gonioscopy	4.45 ± 0.60	2.21 ± 1.14	< 0.001	2.59 ± 1.36
Intraocular pressure	4.87 ± 0.34	4.86 ± 0.38	0.940	4.86 ± 0.37
Optic nerve head	4.7 ± 0.47	4.49 ± 0.57	0.119	4.53 ± 0.56

^a^ Confidence level was rated on a Likert scale: none = 1, slightly = 2, moderately = 3, highly = 4 and extremely = 5

* SD = standard deviation

All the GS felt highly to extremely confident in the assessment of AC depth and ACA, and 21 (91.3%) in dynamic gonioscopy. As could be expected, there was a significant difference between GS and NGS in the confidence in assessment of AC depth (*p* = 0.041) and performing gonioscopy (*p* < 0.001), but not in measuring IOP (*p* = 0.940) or ONH evaluation (*p* = 0.119). We believe this discrepancy stems from the advanced training the GS had received during their glaucoma fellowship.

We asked the respondents to assess whether their confidence level in carrying out examinations is associated with specific aspects of their training during residency. A considerable proportion of them (*n* = 61, 44.8%) firmly believe that lower confidence was closely linked to inadequate guidance on the part of attending physicians, followed by insufficient time spent on learning per se (*n* = 50, 36.8%) and on practical experience (*n* = 46, 33.8%), although not reaching a level of significance (*p* = 0.715, *p* = 0.988, and *p* = 0.238, respectively). The GS did not demonstrate any significant difference in the responses to these queries compared to NGS (*p* = 0.358, *p* = 0.957, and *p* = 0.706, respectively).

### Patterns of practice of various ocular examinations

When asked about basic ocular examination techniques (i.e., the frequency of AS and IOP assessment prior to pupil dilation or gonioscopy), we found that only 46.9% of the NGS respondents perform the correct order of examination compared to 78.3% of the GS (*p* = 0.018).

One-third of the respondents (*n* = 46) reported not performing gonioscopy, even for a patient with anatomical predisposition for acute or chronic angle closure glaucoma (e.g., a phakic patient with a shallow AC). Over one-third (*n* = 48, 35.3%) of the respondents had encountered a patient of theirs who presented with AACG following pupil dilation, which might have been prevented if following a gonioscopic assessment it had been known that the patient was at risk. Notably, there was a highly significant difference between the responses of the GS and NGS respondents (*n* = 1, 4.3% vs. *n* = 45, 39.8%, *p* < 0.001, respectively).

Furthermore, the low rate of performing gonioscopy correlated significantly with a low level of confidence in performing the examination (*p* < 0.001), but not with the setting (hospital compared to a community or private clinic, *p* = 0.534) or the number of years of experience among NGS respondents (*p* = 0.257).

### Perceptions of gonioscopy practice

The respondents were asked what they considered to be the reasons for the infrequent practice of gonioscopy. The most selected responses were insufficient time allotted for the visit (*n* = 80, 58.8%) and lack of ophthalmologists’ experience (*n* = 66, 48.5%) with this procedure. Other notable reasons included lack of basic knowledge (*n* = 36, 26.5%) or equipment (*n* = 34, 25%), regularly seen patients with no history of high IOP after pupil dilation (*n* = 34, 25%), and the estimated low percentage of narrow angles in the general local population (*n* = 33, 24.3%). Only 12 (8.8%) respondents identified the patient’s cooperation as a factor influencing the decision to forego gonioscopy.

## Discussion

Glaucoma is an irreversible blinding disease if left untreated. Therefore early diagnosis and treatment are crucial for the preservation of visual acuity and visual field. Gonioscopy is essential in discerning various glaucoma types, monitoring changes over time and implementing appropriate treatment strategies. Glaucoma surgeries have progressed substantially during the past two decades with the introduction of microinvasive glaucoma surgeries (MIGS) which harness the anatomy and function of the ACA to reduce IOP. However, not all MIGS are appropriate for angle-closure types of glaucoma. Additionally, some of the MIGS are marketed as adjuncts to cataract surgery, which can be performed by any cataract surgeon, not solely by GS. Thus, it is imperative that surgeons possess a foundational understanding of gonioscopy and ACA anatomy. As MIGS gain popularity, an increase in gonioscopy practice is anticipated. However, it has been suggested that the utilization of gonioscopy is declining with the advancement of imaging modalities, such as the ultrasound biomicroscope and anterior segment optical coherence tomography [13]. These modalities cannot show all pathologies in the ACA, such as pigmentation or fine abnormal vessels, they are costly, require a skilled technician and a subsequent review of the results by an ophthalmologist, and they are not readily available. In contrast, gonioscopy is a fast and effective diagnostic examination that is carried out as part of the routine ophthalmology examination.

This survey was designed to clarify the extent to which a simple ocular examination, such as gonioscopy, is routinely performed today, the level of training provided during residency, the associated level of confidence in conducting the examination, and any impediments that may hinder its utilization.

Based upon the findings of our survey, only 66.9% of the responding ophthalmologists routinely perform gonioscopy when indicated, and one-fourth of them are GS. This result agrees with published surveys conducted worldwide, which indicate that 46–80% of the qualified ophthalmologists perform routine gonioscopy, the highest percentage being reported from Singapore, likely due to their high incidence of AACG [14–22]. Documentation of gonioscopy among residents in the USA was reported to be as high as 76.8% in 2005 [19] but as low as 46% one decade later at another center [20]. In Nigeria, 66% of the ophthalmologists report performing gonioscopy although 85% report they have access to an available goniolens [21]. In India, fewer than 50% of the NGS perform gonioscopy [22]. 

When analyzing the responses of the subgroup of GS in our survey, it emerged, as anticipated, that the overwhelming majority (95.7%) of them perform routine gonioscopy, in line with the higher range of 70.8–96.6% reported in the literature [14, 15, 17, 22–24]. These findings illustrate that while GS acknowledge the significance of gonioscopy, NGS tend to disregard it.

Most GS in our survey attribute the limited utilization of gonioscopy among ophthalmologists to lack of experience or knowledge, and associate lower confidence in examination with lack of time allotted to teaching, training and feedback from a senior attending during residency. Furthermore, our findings represent the initial evidence indicating that confidence does not accrue over years of experience through repeated exposure to specific examinations without the basic training. Previous studies mentioned the association between training and confidence but surveyed only residents. Feng et al. (2019) found a low level of trainee satisfaction in the United-Kingdom with both the quantity and quality of gonioscopy teaching, associated with poor confidence in gonioscopy [25]. Gogate et al. (2021) explored the perspectives of residency instructors in India [26]. Their study revealed that while residents were expected to possess a high level of proficiency in gonioscopy, with a mean rating of 9.2 out of 10, the grading of training in gonioscopy as reported by young ophthalmology specialists was 5.7 out of 10 [27]. This discrepancy between the teachers’ perception and actual management of real-life residency is the basis for our call for expedited changes of curriculum.

In an effort to remedy the shortcomings of our residency programs, we suggest that various teaching formats can be employed, such as small-group practice-guided meetings with dry and wet labs, case presentations, more time for hands-on training in the glaucoma clinic, and tutoring with live video at the clinic or operating theaters. Some of these methods have been suggested before [25–27], and it will be interesting to analyze the benefit of each incorporated format. Furthermore, it is necessary to emphasize the importance of correct patient screening in continuing medical education and scientific meetings. Other common reasons for the lower utilization of gonioscopy among our survey participants included lack of sufficient time in the clinic. Despite time constraints in clinical settings, it is essential to dedicate sufficient time for gonioscopy in a routine examination. Choosing appropriate goniolens may assist in shortening the examination time. Although mentioned by just a few respondents, negotiations should be initiated with insurance companies to reimburse gonioscopy, which could help increase its utilization.

At the 2023 World Glaucoma Conference, organized by the World Glaucoma Association, a novel addition was made in the form of a gonioscopy workshop. Although the workshop was limited to 50 attendees, it was completely booked with representatives from 28 different countries, including 22 GS, 15 general ophthalmologists, and 10 ophthalmologists in training. The success of this initiative serves as an inspiring example for conference organizers worldwide, urging them to consider incorporating similar workshops and courses to provide broader exposure to NGS and especially trainees.

There are several limitations to our study. First, as with all survey-based studies, the accuracy of the results may be limited by recall and response biases. Second, the nature of our respondents may introduce sampling bias related to uneven distribution across various subspecialties (with 16.9% of respondents identifying themselves as GS) or geographical employment regions (as 62% of respondents were employed in the central part of the country). Moreover, patients in the retina clinic are usually examined by an ophthalmologist after pupil dilation by the optometrist. Finally, 15 (11%) of the respondents were residents at various stages of their residency, and some of their ratings of the training programs’ quality might have been premature. Repeated statistical analysis without the residents’ responses yielded similar results. Despite these limitations, the relatively high number of respondents and the consistency of our statistical analysis provide support for our findings.

## Conclusions

Our survey findings provide insights into the current state of gonioscopy utilization and its association with residency training. This underutilization appears to be primarily due to inadequate training, lack of confidence among ophthalmologists with varied years of experience, and lack of time allotted to office visits in real-life settings. We wish to highlight the need for revision of the current residency curriculum with focus upon enhancing basic knowledge and clinical skills. Gonioscopy is an ocular evaluation vital to diagnosing and managing a leading cause of irreversible blindness, which is predicted to increase with global aging and population growth worldwide.

## Data Availability

The datasets used and analysed during the current study are available from the corresponding author upon reasonable request.

## Abbreviations

POAG: Primary Open-Angle Glaucoma
PACG: Primary Angle-Closure Glaucoma
ACA: Anterior Chamber Angle
AACG: Acute Angle Closure Glaucoma
AS: Anterior Segment
IOP: Intraocular Pressure
ONH: Optic Nerve Head
MIGS: Microinvasive Glaucoma Surgeries
GS: Glaucoma Specialists
NGS: Non Glaucoma Specialists
