# Correction: Bridging gaps in ophthalmology residency programs: the link between practice, training and confidence in ocular examination and gonioscopy for diagnosing glaucoma, a blinding disease

**DOI:** 10.1186/s12909-024-05726-2

**Published:** 2024-07-16

**Authors:** Ortal Fogel Tempelhof, Daphna Mezad-Koursh, Assaf Hilely, Dan Gaton, Shimon Kurtz

**Affiliations:** 1 Department of Ophthalmology, Sourasky Medical Center, Tel Aviv Israel, affiliated to The Faculty of Medicine, Tel Aviv University, Tel Aviv, Israel; 2 Department of Ophthalmology, Rabin Medical Center, Petah-Tikva, Israel, affiliated to The Faculty of Medicine, Tel Aviv University, Tel Aviv, Israel

Correction: BMC Medical Education (2024) 24:685 10.1186/s12909-024-05665-y

Following publication of the original article [1], the authors identified the below errors:

Incorrect affiliations:

1 Department of Ophthalmology, Faculty of Medicine, Tel Aviv Sourasky Medical Center, Tel Aviv University, 6 Weizmann St, Tel Aviv 6,423,906, Israel.

2 Department of Ophthalmology, Faculty of Medicine, Rabin Medical Center, Tel Aviv University, Tel AvivPetah-Tikva, Israel.

Correct affiliations:

1 Department of Ophthalmology, Sourasky Medical Center, Tel Aviv Israel, affiliated to The Faculty of Medicine, Tel Aviv University, Israel.

2 Department of Ophthalmology, Rabin Medical Center, Petah-Tikva, Israel, affiliated to The Faculty of Medicine, Tel Aviv University, Israel.

Also, the duplicate (mean ± SD) from Table 2 has been removed.

The original article has been corrected.

## References

[1] Fogel, Tempelhof, et al. BMC Med Educ. 2024;24685. 10.1186/s12909-024-05665-y. 10.1186/s12909-024-05665-y PMC11193273 38907194

